# Plant elicitor peptide signalling confers rice resistance to piercing‐sucking insect herbivores and pathogens

**DOI:** 10.1111/pbi.13781

**Published:** 2022-02-18

**Authors:** Wenzhong Shen, Xue Zhang, Jiuer Liu, Kehan Tao, Chong Li, Shi Xiao, Wenqing Zhang, Jian‐Feng Li

**Affiliations:** ^1^ State Key Laboratory of Biocontrol Guangdong Provincial Key Laboratory of Plant Resources School of Life Sciences Sun Yat‐sen University Guangzhou Guangdong China

**Keywords:** brown planthopper, immune stimulator, *Magnaporthe oryzae*, plant elicitor peptide, rice, *Xanthamonas oryzae* pv. *oryzae*

## Abstract

Rice is a staple food crop worldwide, and its production is severely threatened by phloem‐feeding insect herbivores, particularly the brown planthopper (BPH, *Nilaparvata lugens*), and destructive pathogens. Despite the identification of many BPH resistance genes, the molecular basis of rice resistance to BPH remains largely unclear. Here, we report that the plant elicitor peptide (Pep) signalling confers rice resistance to BPH. Both rice *PEP RECEPTOR*s (*PEPR*s) and *PRECURSORs of PEP* (*PROPEPs*), particularly *OsPROPEP3*, were transcriptionally induced in leaf sheaths upon BPH infestation. Knockout of *OsPEPR*s impaired rice resistance to BPH, whereas exogenous application of OsPep3 improved the resistance. Hormone measurement and co‐profiling of transcriptomics and metabolomics in OsPep3‐treated rice leaf sheaths suggested potential contributions of jasmonic acid biosynthesis, lipid metabolism and phenylpropanoid metabolism to OsPep3‐induced rice immunity. Moreover, OsPep3 elicitation also strengthened rice resistance to the fungal pathogen *Magnaporthe oryzae* and bacterial pathogen *Xanthamonas oryzae* pv. *oryzae* and provoked immune responses in wheat. Collectively, this work demonstrates a previously unappreciated importance of the Pep signalling in plants for combating piercing‐sucking insect herbivores and promises exogenous application of OsPep3 as an eco‐friendly immune stimulator in agriculture for crop protection against a broad spectrum of insect pests and pathogens.

## Introduction

As sessile organisms constantly encountering attacks from microbial pathogens and insect herbivores, plants have evolved pattern‐triggered immunity (PTI) and effector‐triggered immunity (ETI) to fend off those opponents (Dangl *et al*., 2013). In PTI, plants employ plasma membrane (PM)‐residing pattern recognition receptors (PRRs) to perceive conserved signature molecules from pathogens or herbivores, which are termed microbe‐ or herbivore‐associated molecular patterns (MAMPs or HAMPs), respectively. Specific plant‐derived molecules, either passively released by invader‐induced damages or actively produced as phytocytokines, can serve as damage/danger‐associated molecular patterns (DAMPs) that function similarly to MAMPs/HAMPs. In ETI, plants deploy intracellular nucleotide‐binding leucine‐rich repeat (NLR) receptors to sense effector proteins, which have been secreted by pathogens or herbivores with an original purpose to suppress PTI. Beyond the framework of PTI and ETI, detailed molecular mechanisms underlying plant resistance to insect herbivores have been scarcely understood in comparison with those to pathogens (Ling *et al*., 2019; Zheng *et al*., 2021).

Rice (*Oryza sativa*) is a staple food crop for billions of the global population and meanwhile an attractive nutrient source for insect herbivores and pathogens. The brown planthopper (BPH, *Nilaparvata lugens* Stål), arguably the most destructive insect pest of rice, annually causes staggering loss of global rice production (Savary *et al.,* 2019). Being a piercing‐sucking insect herbivore, BPH can damage rice plants directly by penetrating the long‐cell blocks of leaf sheaths and ingesting the phloem sap using the stylet (Shi *et al.,* 2021), or indirectly by transmitting viral diseases as an insect vector (Ling and Zhang, 2016). Overuse of chemical pesticides currently remains a major solution for rice farmers to cope with BPH, which, however, can stimulate pesticide tolerance in the pests and adversely influence the biodiversity and food safety (Lu *et al.,* 2017; Way and Heong, 1994). By contrast, fortification of rice immunity promises an effective and eco‐friendly approach for BPH control.

The formation of BPH resistance is a multifaceted process in rice (Cheng *et al.,* 2013a). Although there has been no HAMP/DAMP‐PRR or effector‐NLR paradigm being established in rice‐BPH interactions, significant progress has been made in identifying BPH resistance genes in rice. On the one hand, two BPH resistance gene loci, *Bph3* and *Bph15*, have been characterized to encode PM‐residing lectin receptor‐like kinases OsLecRK1‐OsLecRK3 and OsLecRK, respectively (Cheng *et al.,* 2013b; Liu *et al.,* 2015). OsLecRK can interact with OsADF, an actin depolymerizing factor, and OsRRK1, a receptor‐like cytoplasmic kinase, to positively modulate BPH resistance (Cheng *et al.,* 2013b; Ma *et al.,* 2017). Another BPH resistance gene, *Bph32*, has been identified as a PM‐localized short consensus repeat domain‐containing protein (Ren *et al.,* 2016). These cell‐surface proteins are candidate PRRs or positive regulators of PTI induced by unknown HAMPs/DAMPs during BPH infestation. On the other hand, four BPH resistance genes, *Bph9*, *Bph14*, *Bph18* and *Bph26*, have been found to encode coiled‐coil (CC)‐containing NLR proteins (Du *et al.,* 2009; Ji *et al.,* 2016; Tamura *et al.,* 2014; Zhao *et al*., 2016). BPH14 can form homodimers and interact with transcription factors OsWRKY46 and OsWRKY72, leading to increased accumulation of the two OsWRKYs and subsequent upregulation of defence‐related genes (Hu *et al.,* 2017). These intracellular resistance proteins probably serve as immune receptors for unidentified BPH effectors (Wang *et al.,* 2021). In addition, the BPH resistance gene *Bph6* encodes an exocyst‐localized protein that can interact with the exocyst subunit OsEXO70E1 to promote exocytosis and cell wall consolidation (Guo *et al.,* 2018), while *Bph30* and *Bph40* encode atypical leucine‐rich domain‐containing proteins that mediate BPH resistance by fortifying rice sclerenchyma (Shi *et al.,* 2021). Of note, certain BPH biotypes can rapidly adapt to resistant rice varieties harbouring only a single BPH resistance gene (Ling *et al.,* 2019; Zheng *et al.,* 2021), whereas stacking *Bph14* and *Bph15* to simultaneously enhance PTI and ETI has been shown to potentiate BPH resistance in hybrid rice (Hu *et al.,* 2012). These observations suggest that the combination of multilayered anti‐BPH strategies enables more efficient and sustainable BPH resistance in rice.

Plant elicitor peptides (Peps) are a class of phytocytokines produced by a wide range of plant species (Huffaker *et al.,* 2006). The majority of *PRECURSOR*s *of PEP* (*PROPEP*s) can be transcriptionally induced upon pathogen infection or wounding (Bartels and Boller, 2015). Mature Peps are liberated from the C‐termini of PROPEPs through metacaspase‐mediated cleavage and relocated to the apoplast (Hander *et al.,* 2019; Shen *et al.,* 2019), where they are perceived by Pep receptors (PEPRs) to elicit typical PTI responses (Yamaguchi *et al.,* 2006, 2010). Recently, the function of Pep‐PEPR signalling has been expanded to defending chewing insect herbivores. In maize (*Zea mays*), the expression of *ZmPROPEP3* was upregulated upon exposure to *Spodoptera exigua* oral secretions (OS) containing potential HAMPs. Exogenous application of ZmPep3 induced the expression of herbivory defence‐related genes and production of jasmonic acid (JA), ethylene, and phytoalexins, leading to constrained growth of *S. exigua* larva on maize leaves (Huffaker *et al.,* 2013). In Arabidopsis (*Arabidopsis thaliana*), the expression of *AtPROPEP3* and *AtPEPR1*/*AtPEPR2* was strongly induced upon *Spodoptera littoralis* feeding. The Pep‐insensitive *atpepr1pepr2* null plants exhibited decreased JA accumulation in response to *S*. *littoralis* OS and compromised insect resistance (Klauser *et al.,* 2015). In rice, transcriptional activation of *OsPROPEP3* was stimulated by *Mythimna loreyi* OS. Simultaneous treatment of OsPep3 and *M. loreyi* OS elicited stronger anti‐herbivore defence than separate treatments, while overexpression of *OsPEPR1* also augmented defence responses induced by OsPeps or *M. loreyi* OS (Shinya *et al.,* 2018). However, it remains unknown whether the Pep‐PEPR signalling is crucial for plants to defend piercing‐sucking insect herbivores, which have a distinct feeding behaviour and generally induce much less wounding than chewing insects (Du *et al.,* 2009).

In this study, we found that *OsPEPR*s and most *OsPROPEP*s were transcriptionally induced by BPH infestation in rice leaf sheaths. Knockout of *OsPEPR*s impaired rice resistance to BPH, whereas exogenous application of OsPep3 exerted an opposite effect. We further conducted hormone measurement and joint profiling of transcriptomics and metabolomics using OsPep3‐elicited rice leaf sheaths to obtain molecular clues as to how the Pep‐PEPR signalling contributes to rice immunity. In addition, OsPep3 application also improved rice resistance to devastating fungal blast disease and bacterial blight disease and was capable of inducing immune responses in wheat. This work demonstrates the importance of Pep signalling in plants for defending piercing‐sucking insect herbivores and inspires the use of OsPep3 as a widely useful crop immune stimulator in agriculture against a broad spectrum of insect pests and pathogens.

## Results

### OsPEPRs positively regulate rice resistance to BPH

**Figure 1:**
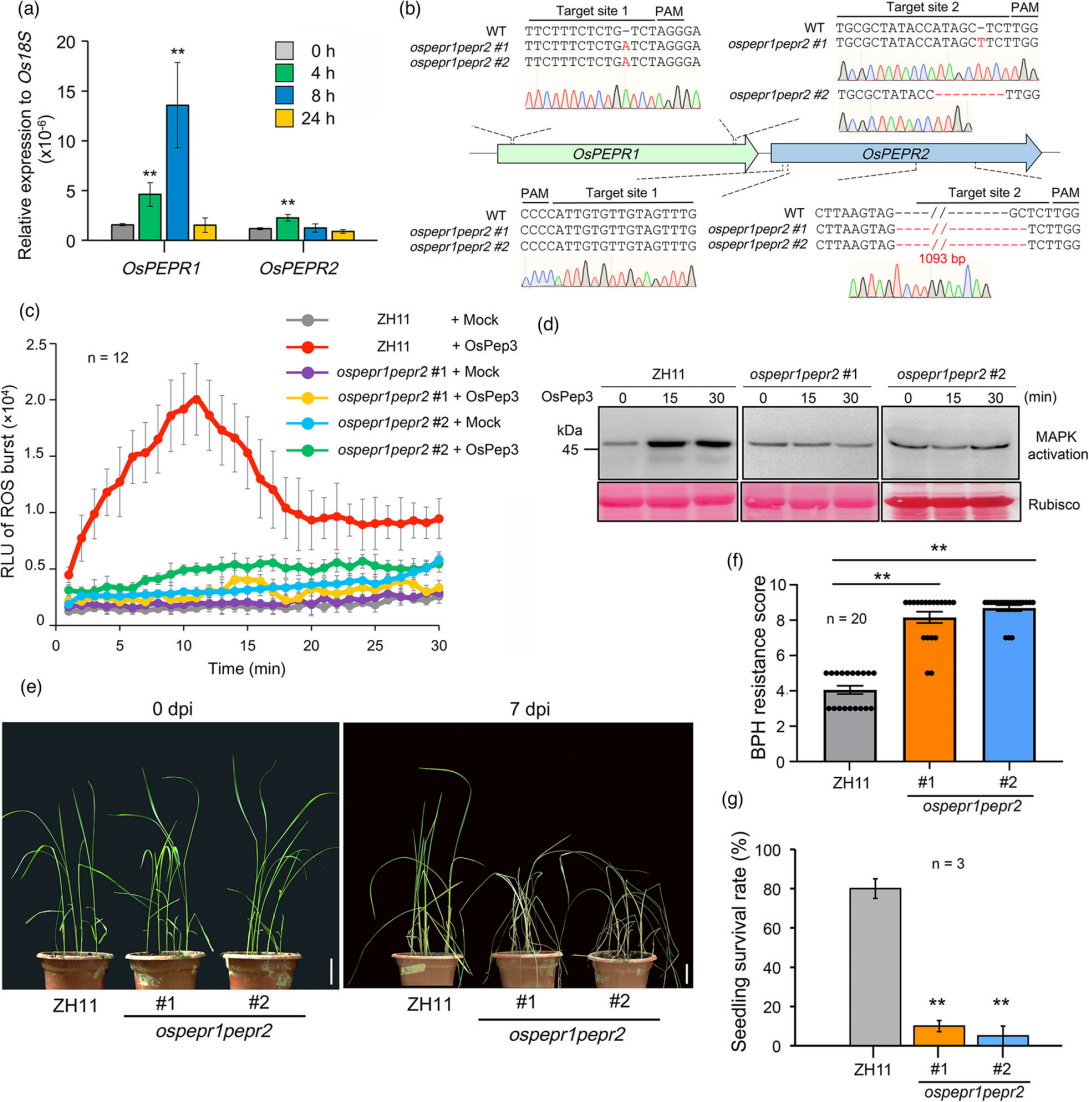
OsPEPRs positively regulate rice resistance to BPH. (a) *OsPEPR*s can be transcriptionally induced in rice leaf sheaths by BPH infestation. Expression levels of *OsPEPR*s were determined by RT–qPCR using *Os18s* as a reference gene. Data are shown as mean ± SD of three biological replicates. **P < 0.01 (Student’s *t*–test). (b) Schematic diagram of CRISPR–mediated knockout of *OsPEPR1* and *OsPEPR2*. Indels or genomic deletions introduced by CRISPR are highlighted in red. (c) OsPep3–induced ROS burst is abolished in *ospepr1pepr2* null plants. ROS burst was determined for 12 leaves of 10–day–old rice seedlings using the luminol–based method. (d) OsPep3–induced MAPK activation is abolished in *ospepr1pepr2* null plants. MAPK activation was determined by immunoblotting using anti–ERK antibodies. Rubisco staining indicates equal loading of total proteins. (e) The *ospepr1pepr2* null plants are more susceptible than ZH11 plants to BPH infestation. Rice seedlings at the four–leaf stage were infested with BPH nymphs of the third instar at a density of 15 insects per plant for 7 days. (f) Quantification of the BPH resistance scores for different genotypes. Twenty seedlings were evaluated for each genotype. Data are shown as mean ± SD with individual data points shown as dots. **P < 0.01 (Student’s *t*–test). (g) The *ospepr1pepr2* null plants have lower survival rates than ZH11 plants under BPH infestation. Data are shown as mean ± SD of three biological replicates. **P < 0.01 (Student’s *t*–test).

The initial goal of this study was to use the rice‐BPH interaction as a model to understand whether the PEPR‐mediated Pep signalling is important for defending piercing‐sucking insect herbivores in plants. The rice genome encodes two *PEPR* genes, namely *OsPEPR1* and *OsPEPR2* (Shinya *et al.,* 2018). To quickly assess the functional relevance of *OsPEPR1* and *OsPEPR2* to BPH resistance, we evaluated the time‐course induction of these genes by BPH infestation in rice leaf sheaths. A substantial increase in *OsPEPR1* expression was observed at 4 and 8 h post‐BPH infestation (hpi), whereas its expression was restored to the basal level at 24 hpi (Figure 1a). By contrast, BPH challenge induced a modest increase in *OsPEPR2* transcripts at 4 hpi, which declined back to the basal level at as early as 8 hpi (Figure 1a). These results hinted that *OsPEPR1* and *OsPEPR2* may play major and minor roles, respectively, in defending BPH.

The function of OsPEPRs in perceiving OsPep3 has recently been supported using *OsPEPR*‐overexpressing plants (Shinya *et al.,* 2018). However, the loss‐of‐function evidence has been lacking. We utilized the CRISPR/Cas9 technology to generate *ospepr1pepr2* null plants in the Zhonghua 11 (ZH11) background and subsequently analysed the alteration of OsPep3 responses in these mutants. As *OsPEPR1* and *OsPEPR2* are located adjacently in the rice genome, we designed four single guide RNAs (sgRNAs), two for each gene, to increase the possibility of large genomic deletions spanning *OsPEPR*s. However, we only obtained two homozygous *ospepr1pepr2* null alleles (i.e. #1 and #2; Figure 1b) containing the same insertion of 1 base pair (bp) at the target site 1 of *OsPEPR1*, leading to a premature stop codon, and the same deletion of 1093 bp at the target site 2 of *OsPEPR2*, leading to the loss of entire kinase domain. The two double null alleles were differed by an additional mutation at the target site 2 of *OsPEPR1*, which was either an insertion of 1 bp in the line #1 or deletion of 8 bp in the line #2 (Figure 1b). The sgRNA aiming at the target site 1 of *OsPEPR2* somehow failed to introduce any mutation (Figure 1b).

The two *ospepr1pepr2* null alleles were morphologically indistinguishable from ZH11 plants at the seedling and heading stages (Figure S1a–f). However, when the leaves of 10‐day‐old ZH11 plants were treated with OsPep3, we could readily detect typical immune responses, including the burst of reactive oxygen species (ROS) and activation of mitogen‐activated protein kinases (MAPKs), at as early as 15 min after OsPep3 exposure (Figure 1c,d). By contrast, the OsPep3‐elicited ROS burst and MAPK activation was abolished in *ospepr1pepr2* alleles (Figure 1c,d). To investigate the physiological role of OsPEPRs in BPH resistance, we fed ZH11 and *ospepr1pepr2* seedlings at the four‐leaf stage to BPH nymphs of the third instar at a density of 15 insects per plant. The *ospepr1pepr2* plants appeared to be more susceptible to BPH infestation than ZH11 plants (Figure 1e). The susceptibility was further quantified by the resistance score (Figure 1f), according to the routinely used criteria (Figure S2) (Huang *et al.,* 2001), and by the plant survival rate (Figure 1g). Notably, in a host choice test where ZH11 and *ospepr1pepr2* plants were grown side‐by‐side, we failed to detect any significant difference between the numbers of insects settled on these two genotypes at 48 hpi (Figure S3), excluding the possibility that the susceptibility of *ospepr1pepr2* plants is due to increased attractiveness to the pests. Moreover, there was no obvious difference in the honeydew weight and body weight gain between BPH nymphs feeding on ZH11 or *ospepr1pepr2* plants at 48 hpi (Figure S4a,b), suggesting that the pests feed equally on the two rice genotypes. Taken together, these findings indicated that the presence or absence of OsPEPRs affects rice resistance to BPH.

### Rice genome encodes seven PROPEPs

**Figure 2:**
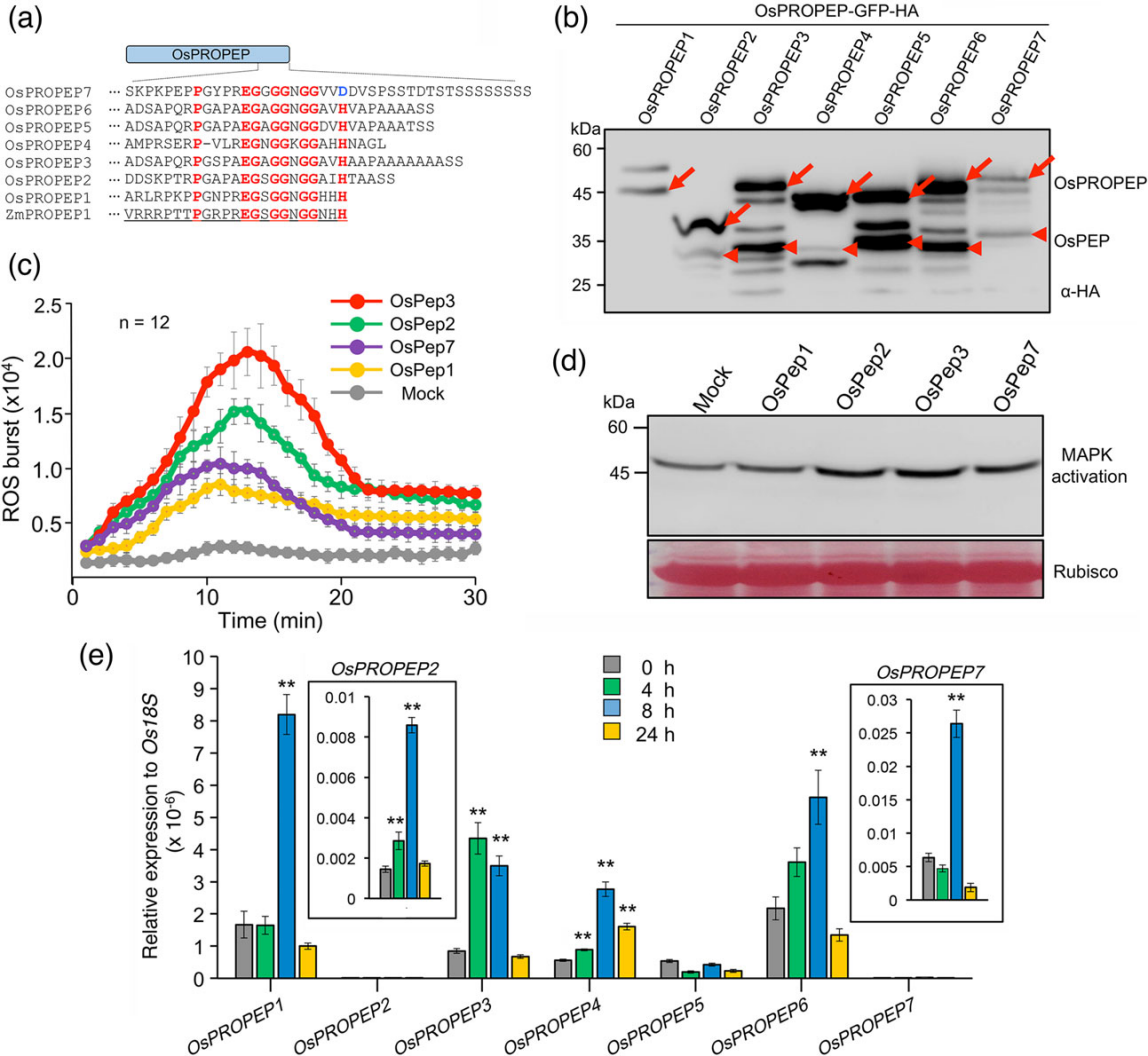
Exogenous application of OsPeps elicits immune responses in rice. (a) OsPep7 exhibits sequence similarity to other OsPeps. The conserved residues of OsPeps are highlighted in red, and the non–conserved ending residue of OsPep7 is marked in blue. ZmPep1 is underlined. (b) OsPROPEPs undergo spontaneous cleavage in protoplasts to produce OsPeps with expected sizes except OsPROPEP1. OsPROPEPs were expressed in rice protoplasts for 12 h. OsPROPEPs and OsPeps are indicated by arrows and arrowheads, respectively. (c) Exogenous application of different OsPeps can elicit ROS burst to varying levels in rice. ROS burst was determined for 12 leaves of 10–day–old rice seedlings using the luminol–based method. (d) Exogenous application of different OsPeps can elicit MAPK activation to varying levels in rice. MAPK activation was determined by immunoblotting using anti–ERK antibodies. Rubisco staining indicates equal loading of total proteins. (e) *OsPROPEP*s can be transcriptionally induced by BPH infestation except *OsPROPEP5*. Expression levels of *OsPEPR*s were determined by RT–qPCR using *Os18s* as a reference gene. Data are shown as mean ± SD of three biological replicates. **P < 0.01 (student’s *t*–test).

We next sought to consolidate the importance of the Pep‐PEPR signalling for BPH resistance from the angle of OsPeps. In an earlier study by Lori and colleagues, three *OsPROPEP*s have been identified in rice (Lori *et al.,* 2015). This gene family has recently been updated to six members (i.e. *OsPROPEP1*‐*OsPROPEP6*) (Shinya *et al.,* 2018). By conducting the BLAST analysis of rice genome using the sequence of ZmPep1 (Huffaker *et al.,* 2011), we identified a new member named OsPROPEP7 (encoded by Os08g0173400), which has a varying and elongated C‐terminal sequence but encompasses a fragment (i.e. OsPep7) showing sequence similarity to the putative OsPep1‐OsPep6 (Figure 2a). A phylogenetic analysis based on predicted Peps from 20 plant species out of four families indicated that Peps from the same plant family are more closely related in sequence (Figure S5). Similar conclusions have been made by two studies conducting phylogenetic analyses using full‐length PROPEPs (Lori *et al.,* 2015; Poretsky *et al.,* 2020). Interestingly, we noticed two exceptions, namely Arabidopsis AtPep6 and *Brassica rapa* BrPep5, which resemble Solanaceous Peps more than Brassicaceous Peps (Figure S5).

We have recently reported that most AtPROPEPs produced in Arabidopsis protoplasts can be spontaneously cleaved by type‐II metacaspases (Shen *et al.,* 2019). When OsPROPEP1‐OsPROPEP7 were expressed in rice protoplasts with C‐terminal GFP‐HA dual tags to allow the detection of processed Peps through immunoblotting, we noted that all OsPROPEPs could be cleaved to generate Pep‐GFP‐HA fragments with deduced sizes (i.e. ~35 kDa), except OsPROPEP1 (Figure 2b). These results suggested that most OsPROPEPs are able to release OsPeps in rice cells in a similar manner as AtPROPEPs in Arabidopsis.

The newly identified OsPep7 does not have the conserved ending histidine residue like OsPep1‐OsPep6 (Figure 2a) and has not been biochemically validated. Therefore, we compared the eliciting activity of OsPep7 with those of OsPep1, OsPep2 and OsPep3. When the leaves of 10‐day‐old ZH11 plants were treated with individual OsPeps, OsPep3 was found to elicit the strongest ROS burst and MAPK activation, while OsPep2 and OsPep7 could induce intermediate levels of immune responses (Figure 2c,d). Notably, OsPep1 only exhibited a moderate eliciting activity (Figure 2c,d). These results verified OsPep7 as a bioactive peptide and OsPep3 as one of the most potent OsPeps.

### OsPep3 treatment improves rice resistance to BPH

**Figure 3:**
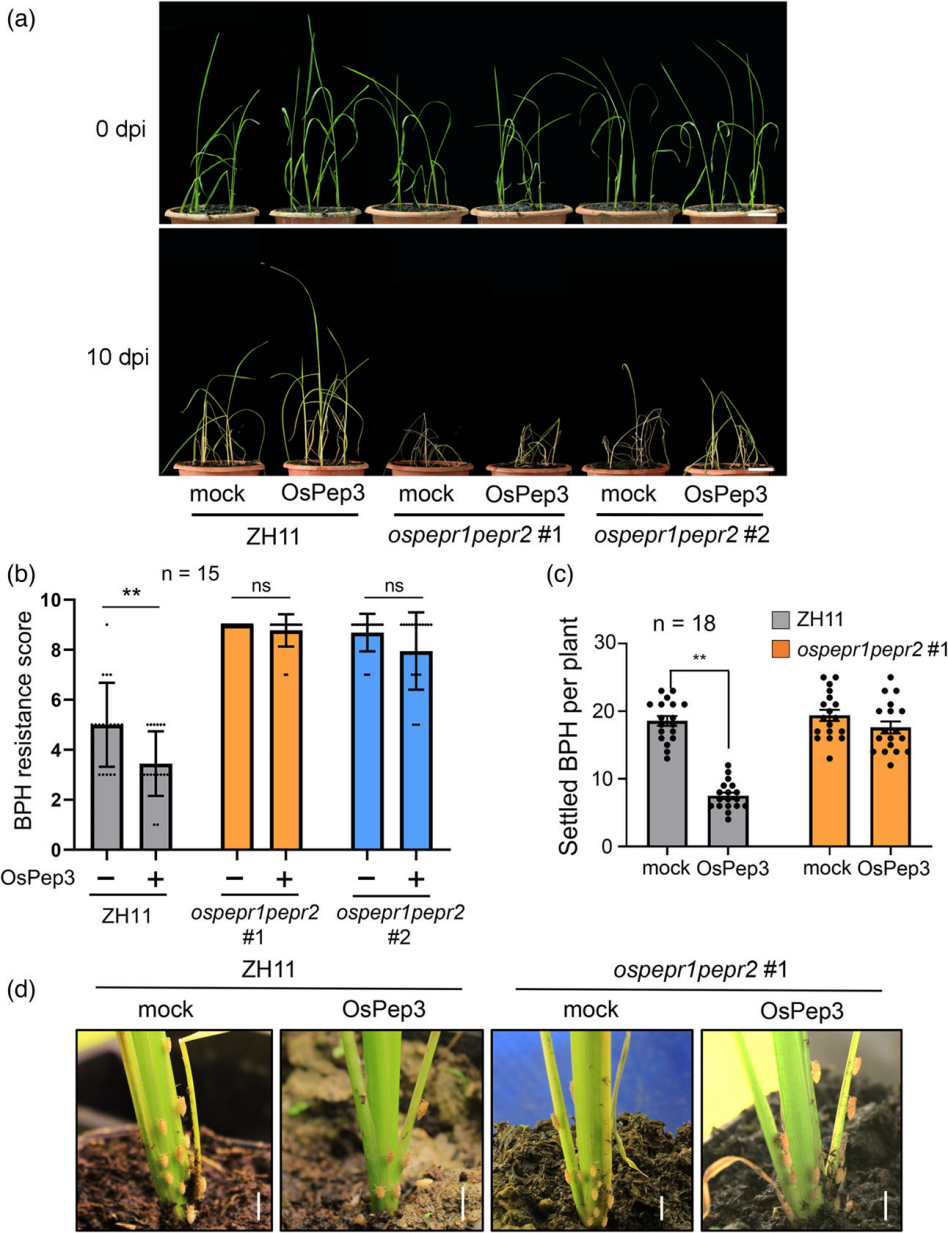
Exogenous application of OsPep3 boosts rice resistance to BPH. (a) OsPep3 treatment enhances BPH resistance in ZH11 but not in *ospepr1pepr2* null plants. Rice seedlings at the four–leaf stage were pretreated with OsPep3 and infested with BPH nymphs of the third instar at a density of 15 insects per plant at 12 h after OsPep3 pretreatment. Plants under BPH attacks were sprayed with OsPep3 every 24 h for 10 days before photographing. Scale bar = 2 cm. dpi, day post–infestation. (b) Quantification of the BPH resistance score for different genotypes. Fifteen seedlings were evaluated for each genotype. Data are shown as mean ± SD with individual data points shown as dots. **P < 0.01 (Student’s *t*–test). (c) BPH prefers to feed on OsPep3–unelicited ZH11 plants in a host choice test. The number of nymphs on each plant was recorded at 48 h post–infestation. Eighteen seedlings were evaluated for each genotype. Data are shown as mean ± SD with individual data points shown as dots. **P < 0.01 (Student’s *t*–test). (d) Representative image for (c). Scale bar = 0.5 cm.

We noted that all *OsPROPEP*s, except *OsPROPEP5*, could be transcriptionally upregulated in rice leaf sheaths under BPH challenge (Figure 2e). Of note, only *OsPROPEP3* had a decent basal expression and meanwhile was highly induced by BPH infestation at 4 hpi (Figure 2e). By contrast, *OsPROPEP7* was still expressed at relatively low levels before and after BPH infestation (Figure 2e). Based on these findings and the earlier observation that OsPep3 could potently induce rice immune responses (Figure 2c,d), we examined whether OsPep3 application can boost rice resistance to BPH. To this end, ZH11 and *ospepr1pepr2*#1 plants at the four‐leaf stage were pretreated with OsPep3 or mock. At 12 h post‐pretreatment, these plants were fed to 15 BPH nymphs of the third instar per plant. In 10 days with a single spray of OsPep3 every 24 h, we noted that the OsPep3‐treated ZH11 plants displayed increased BPH resistance relative to mock‐treated plants (Figure 3,b). By contrast, the *ospepr1pepr2* plants exhibited impaired basal resistance to BPH, while OsPep3 treatment was unable to improve the resistance (Figure 3a,b), which is consistent with our earlier finding that the *ospepr1pepr2* plants were insensitive to OsPep3 (Figure 1c,d). Moreover, reduced insects were found to settle on OsPep3‐treated ZH11 plants relative to mock‐treated plants at 48 hpi in a host choice test (Figure 3c,d), suggesting that exogenous application of OsPep3 can mobilize the antixenosis defence mechanism in rice against BPH. In the same test, ZH11 and *ospepr1pepr2* plants showed no difference in the number of insects settled on individual plants (Figure 3c,d), which is in agreement with our earlier result (Figure S3). We also checked the honeydew weight and body weight gain of BPH nymphs feeding on OsPep3‐ or mock‐treated ZH11 plants at 48 hpi. The insects feeding on OsPep3‐treated ZH11 plants had reduced honeydew weight and body weight gain when compared with those feeding on mock‐treated ZH11 plants (Figure S6a,b), suggesting that OsPep3‐elicited rice plants can reduce the feeding of BPH. These findings further bolstered the notion that the Pep‐PEPR signalling plays a critical role in rice for defending BPH.

### OsPep3 treatment induces JA accumulation in rice

**Figure 4:**
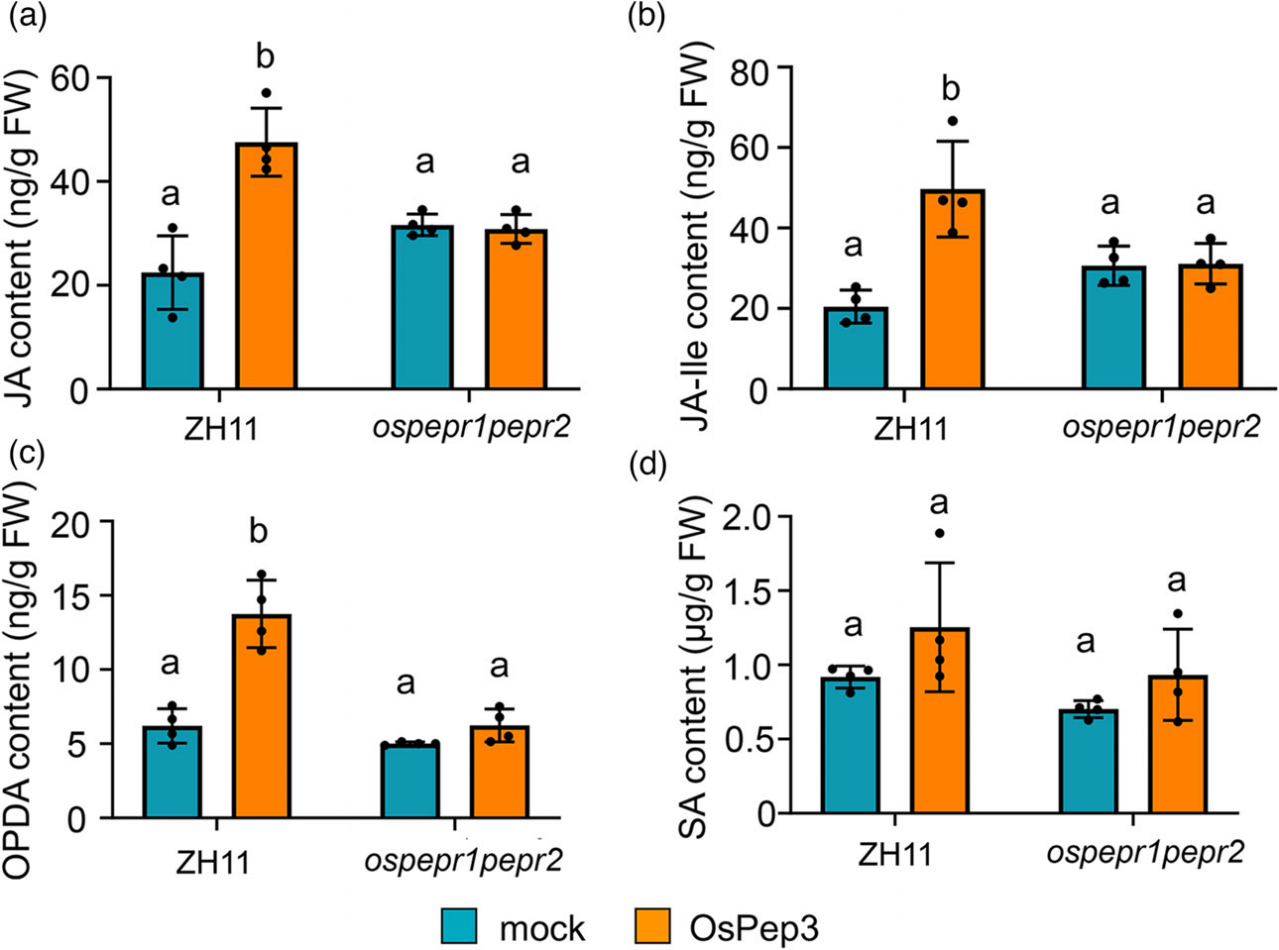
Exogenous application of OsPep3 elicits the accumulation of JA and OPDA in rice leaf sheaths. (a–c) OsPep3 induces the accumulation of JA (a), JA–Ile (b) and OPDA (c) in ZH11 but not in *ospepr1pepr2* null plants at 24 h post–treatment. (d) OsPep3 cannot induce a significant change in SA abundance in ZH11 plants at 24 h post–treatment. For (a), (b), (c) and (d), data are shown as mean ± SD with individual data points shown as dots. Different letters indicate significant differences with *P* < 0.01 (one–way anova with Tukey’s multiple comparisons test).

Both salicylic acid (SA) and JA have been assigned with important roles in rice resistance to BPH (Du *et al.,* 2009; He *et al.,* 2020; Xu *et al.,* 2021). In addition, 12‐oxo‐phytodienoic acid (OPDA), an intermediate metabolite in JA biosynthesis, has also been shown to stimulate rice resistance to BPH (Guo *et al.,* 2014). Therefore, we evaluated OsPep3‐induced changes in the abundances of JA, JA‐isoleucine (JA‐Ile, the bioactive form), OPDA, and SA in the leaf sheaths of ZH11 and *ospepr1pepr2* plants. At 24 h post‐treatment, the OsPep3‐treated ZH11 plants were found to accumulate significantly more JA, JA‐Ile and OPDA than mock‐treated plants (Figure 4a–c), whereas no statistically significant difference in SA abundance was detected between OsPep3‐ and mock‐treated plants (Figure 4d). These results implicated that the JA pathway contributes to OsPep3‐induced BPH resistance in rice.

### OsPep3 treatment induces transcriptional reprogramming in rice

**Figure 5:**
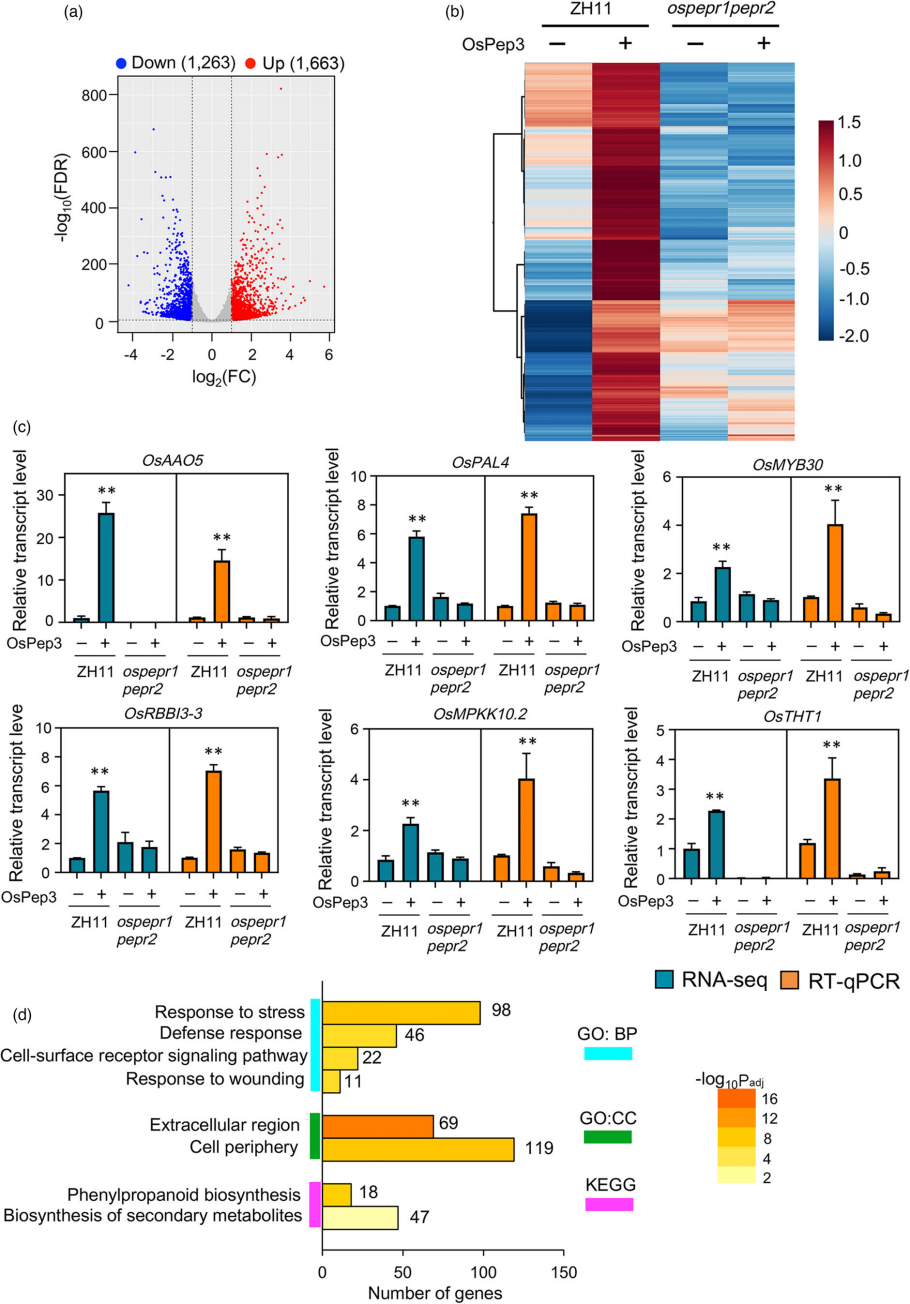
OsPep3 elicits transcriptional reprogramming in rice leaf sheaths. (a) Volcano plot displays differentially regulated genes (DEGs) with fold change (FC) > 2 and *P* < 0.01 at 24 h after OsPep3 treatment in two biological replicates. FDR, false discovery rate. (b) Hierarchical clustering of OsPep3–upregulated genes at 24 h after OsPep3 treatment. Expression levels of each gene across different samples are shown as *Z*–scores scaled by the FPKM (fragments per kilobase of transcript per million mapped reads). (c) RT–qPCR validation (orange) of RNA–seq data (blue) using six OsPep3–upregulated genes. Expression levels of these genes in ZH11 plants at 24 h after mock treatment were normalized as 1. RT–qPCR data are shown as mean ± SD of three biological replicates. **P < 0.01 (Student’s *t*–test). (d) GO and KEGG analyses of OsPep3–upregulated genes. BP, biological process. CC, cellular compartment. Padj, adjusted *P* value.

To obtain more molecular clues about OsPep3‐induced BPH resistance in rice, we conducted RNA‐sequencing (RNA‐seq) analysis using the leaf sheaths of ZH11 or *ospepr1pepr2* plants at 24 h after OsPep3 or mock treatment. We detected 1,663 upregulated and 1,263 downregulated genes (fold change > 2) induced by OsPep3 in ZH11 plants (Figure 5a) (see Sequence Read Archive database, accession ID: PRJNA766326). We chose to focus on the OsPep3‐upregulated genes since they may provide straightforward mechanistic explanation on the OsPep3‐induced BPH resistance. Among these genes, 723 genes with higher basal expression levels in *ospepr1pepr2* plants were excluded from our attention, considering that the *ospepr1pepr2* plants were more susceptible to BPH attacks than ZH11 plants (Figure 1e–g). For the remaining genes (Figure 5b), we validated the robustness of the RNA‐seq data by selecting a few defence‐related genes and evaluating their OsPep3‐inducible patterns using quantitative reverse transcription PCR (RT‐qPCR) (Figure 5c). The 940 upregulated genes were then subjected to gene ontology (GO) and Kyoto encyclopaedia of genes and genomes (KEGG) analyses. The GO analysis showed that stress responses, defence responses, cell‐surface receptor signalling and wounding responses were major biological processes (BPs) stimulated by OsPep3, while cellular components mobilized by OsPep3 were overrepresented by those located at the extracellular region and cell periphery (Figure 5d). Moreover, the KEGG analysis highlighted the engagement of many OsPep3‐upregulated genes in the biosynthesis of secondary metabolites, particularly the phenylpropanoid pathway (Figure 5d).

We also looked into individual OsPep3‐induced genes in ZH11 plants with functional annotations. Notably, among the top 15 genes induced by OsPep3 (Figure S7), three genes, Os01g0822900 (*OsLTP1.2*), Os06g0604200 (*OsPLDα4*) and Os03g0167000 (*OsLTPG2*), are involved in lipid metabolism, while four genes, Os04g0107600 (*OsADC2*), Os07g0526400 (*OsPKS15*), Os09g0507300 (*OsAAO5*) and Os08g0140300 (*OsTDC1*), encode enzymes for the biosynthesis of secondary metabolites. In particular, a role of ascorbate oxidase, encoded by *OsAAO5* (Figure 5c and Figure S7), in defending insect herbivores has been proposed (Felton and Summers, 1993). Beyond the top 15, *OsMYB30* and *OsPAL4*, two players exerting positive effects on BPH resistance through promoting phenylpropanoid biosynthesis (He *et al.,* 2020), were found to be upregulated by OsPep3 (Figure 5c). These results suggested potential roles of lipid metabolism and biosynthesis of secondary metabolites, especially phenylpropanoids, in OsPep3‐induced rice resistance to BPH.

### OsPep3 treatment elicits metabolomic changes in rice

**Figure 6:**
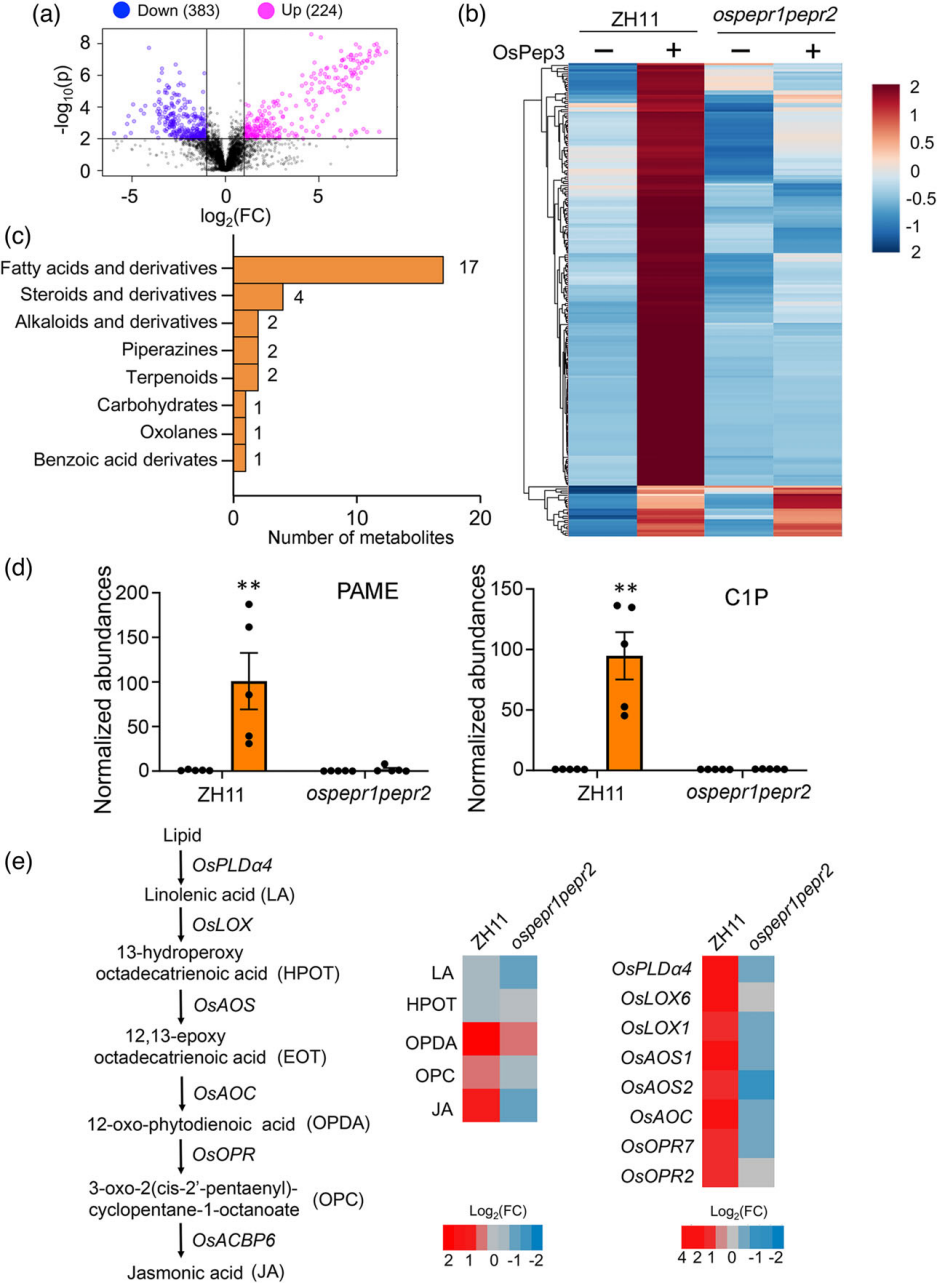
OsPep3 elicits metabolic changes in rice leaf sheaths. (a) Volcano plot displays differentially produced metabolites with fold change (FC) > 2 and *P* < 0.01 at 24 h after OsPep3 treatment in five biological replicates. (b) Hierarchical clustering of OsPep3–upregulated metabolites at 24 h post–treatment. The abundances of each metabolite across different samples are shown as *Z*–scores of their intensity in the metabolome. (c) Fatty acids and derivatives are overrepresented in OsPep3–upregulated metabolites. (d) Palmitelaidic acid methyl ester (PAME) and ceramide–1–phosphate (C1P) represent most highly inducible metabolites by OsPep3. Data are shown as mean ± SD of five biological replicates. **P < 0.01 (Student’s *t*–test). (e) Joint profiling of metabolomics (left heatmap) and transcriptomics (right heatmap) indicates that the JA biosynthetic pathway is upregulated by OsPep3. The induction fold of each metabolite/gene by OsPep3 is indicated by the colour scale based on the log_2_ fold change.

In parallel to the transcriptome profiling, we also harnessed the same batch of leaf sheath tissues to perform non‐targeted metabolome analysis. We detected 224 and 383 metabolites with increased or decreased abundances (fold change > 2), respectively, in ZH11 plants at 24 h post‐OsPep3 treatment (Figure 6a) (see MetaboLights database, accession ID: MTBLS3518). We focused on the 224 metabolites upregulated by OsPep3 (Figure 6b), since they may have a causal relationship with the OsPep3‐induced BPH resistance. Unfortunately, only 30 of these metabolites have been annotated thus far (Figure S8a). Intriguingly, fatty acids and derivatives, which are extensively involved in plant immunity (Lim *et al.,* 2017), were overrepresented among OsPep3‐upregulated metabolites (Figure 6c). Of note, palmitelaidic acid methyl ester (PAME) and ceramide‐1‐phosphate (C1P) were ranked the first and the second, respectively, among all annotated metabolites induced by OsPep3 (Figure 6d and Figure S8b). PAME is a variant of palmitoleic acid, an unsaturated fatty acid that has been shown to induce defence priming against insect herbivores (Li *et al.,* 2016). C1P is a bioactive phosphorylated sphingolipid that positively regulates cell survival and defence responses (Bi *et al.,* 2014). These findings hinted that fatty acids and derivatives may be key players in OsPep3‐induced BPH resistance in rice.

By jointly analysing OsPep3‐induced transcriptomic and metabolomic changes in rice, we observed a consensus regarding the upregulation of JA biosynthesis by OsPep3. At the transcript level, most of the genes involved in JA biosynthesis have been upregulated by OsPep3, including *OsPLDα4*, *OsLOX1/6*, *OsAOS1/2*, *OsAOC* and *OsOPR2/7* (Figure 6e). At the metabolic level, the abundances of JA and two intermediate metabolites in its biosynthetic pathway, namely OPDA and 3‐oxo‐2(cis‐2‐pentenyl)‐cyclopentane‐1‐octanoic acid (OPC), were increased to different levels upon OsPep3 elicitation (Figure 6e). These results echoed our earlier observations based on hormone measurements (Figure 4a–c) and supported the notion that the JA pathway contributes to OsPep3‐induced rice resistance to BPH.

### Expanded use of OsPep3 in crop protection against fungal and bacterial pathogens

**Figure 7:**
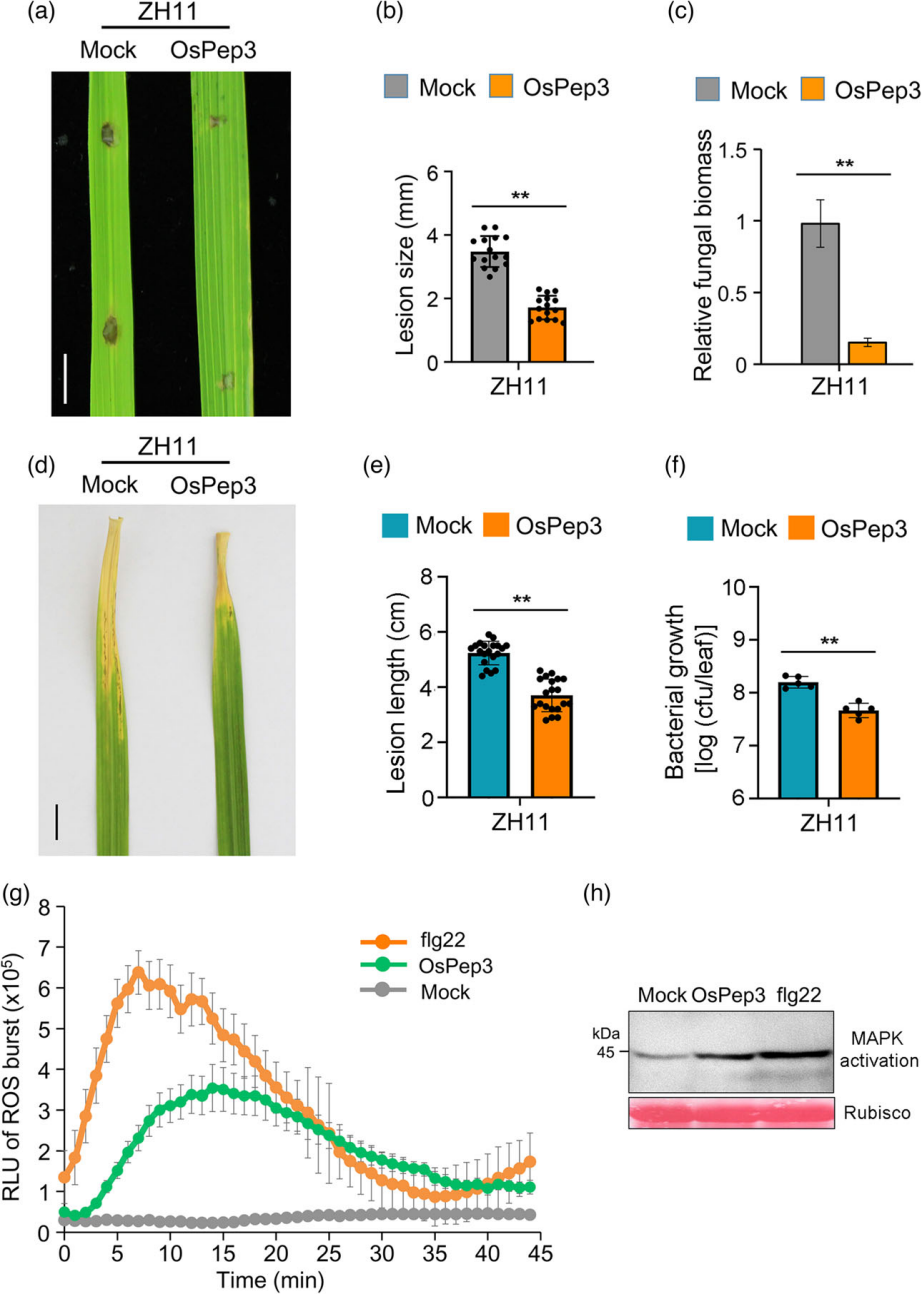
Expanded application of OsPep3 in crop protection. (a) OsPep3 treatment enhances *Magnaporthe oryzae* resistance in ZH11 plants. Detached leaves of 1–month–old ZH11 plants were pretreated with OsPep3 or mock for 24 h and inoculated with 10^3^ conidia in the presence of OsPep3 for 7 days. Scale bar = 1 cm. (b) Quantification of lesion sizes. Data are shown as mean ± SD of 15 lesions with individual data points shown as dots. **P < 0.01 (Student’s *t*–test). (c) Quantification of fungal biomass around the infection site. Quantification was based on the qPCR assay of the abundance of fungal Pot2 DNA against that of rice ubiquitin DNA. Data are shown as mean ± SD of three biological replicates. **P < 0.01 (Student’s *t*–test). (d) OsPep3 treatment enhances *Xanthamonas oryzae* pv. *oryzae* (*Xoo*) resistance in ZH11 plants. Two–month–old rice plants were inoculated with *Xoo* at OD_600_ of 0.6 using the leaf–clipping method. OsPep3 or mock was applied by spraying at 12 h before bacterial inoculation and every 12 h after inoculation. Rice blight symptoms were recorded 10 days after inoculation. (e) Quantification of lesion lengths in *Xoo*–infected leaves. Data are shown as mean ± SD of 20 lesions with individual data points shown as dots. **P < 0.01 (Student’s *t*–test). (f) Quantification of bacterial growth in *Xoo*–infected leaves. Data are shown as mean ± SD of 5 leaves with individual data points shown as dots. **P < 0.01 (Student’s *t*–test). cfu, colony–forming unit. (g) Exogenous application of OsPep3 can elicit ROS burst in wheat. ROS burst was determined for 12 leaves of 14–day–old wheat seedlings using the luminol–based method. flg22 was used as a positive control. (h) Exogenous application of OsPep3 can elicit MAPK activation in wheat. MAPK activation was determined by immunoblotting using anti–ERK antibodies. Rubisco staining indicates equal loading of total proteins.

Many OsPep3‐induced genes, such as *OsPAL4*, *OsMYB30*, *OsRBBI3‐3*, *OsMAPKK10.2* and *OsTHT1* (Figure 5c), have been assigned additional roles in rice resistance to pathogens (Chen *et al.,* 2014; Li *et al.,* 2020; Ma *et al.,* 2021; Shen *et al.,* 2021; Tonnessen *et al.,* 2015), which prompted us to test whether OsPep3 application also improves rice resistance to pathogens. Indeed, we found that OsPep3‐treated ZH11 plants were more resistant than mocked‐treated plants to the fungal pathogen *Magnaporthe oryzae*, the causal agent of rice blast disease, according to the lesion size and fungal biomass around the infection site (Figure 7a–c). In addition, OsPep3 treatment also rendered ZH11 plants more resistant than untreated plants to the bacterial pathogen *Xanthamonas oryzae* pv. *oryzae* (*Xoo*), the causal agent of rice blight disease, based on the lesion length and bacterial colony counting of infected leaves (Figure 7d–f). These results indicated that exogenous application of OsPep3 also fortifies rice resistance to devastating diseases.

To date, no Pep has been functionally characterized in wheat. We were wondering whether OsPep3 application is effective for stimulating immune responses in wheat. We found that OsPep3 treatment could also activate ROS burst and MAPK in wheat (Figure 7g,h), albeit to a lower level than those activated by flg22, a potent immunogenic peptide of bacterial flagellin. These findings suggested that OsPep3 is a widely effective immune stimulator for Poaceae crop plants.

## Discussion

Although plant defence mechanisms against microbial pathogens have been extensively illustrated, how plants ward off insect foes has been poorly understood, particularly for piercing–sucking insect herbivores (Qi *et al.,* 2018). Unlike chewing herbivores that cause enormous wounding on plant tissues, piercing–sucking insects feed on the phloem sap using the stylet, which minimizes the wounding of host tissues and reduces the contact of plant cells to the insect saliva containing potential HAMPs. This cunning feeding strategy has posed great challenges on the host plants to develop timely and effective defence. Indeed, for BPH, a rice–specific piercing–sucking insect pest, only a mucin–like protein from its saliva has been identified so far as an elicitor of rice immune responses (Shangguan *et al.,* 2018). On the other hand, plants have evolved a smart countermeasure to amplify the invader–induced immunity by producing phytocytokines (Hou *et al.,* 2021). Systemin, the first known phytocytokine, has been found to promote defence responses in Solanaceous plants against the chewing insect herbivore *Manduca sexta* (Orozco–Cardenas *et al.,* 1993). Later, another class of wounding–induced phytocytokines, plant Peps, has been reported with central roles in defending chewing insect pests (Huffaker *et al.,* 2013; Klauser *et al.,* 2015; Shinya *et al.,* 2018). Unlike systemin that exclusively exists in the Solanaceous family, plant Peps are ubiquitously present across the plant kingdom (Bartels and Boller, 2015), thus bearing increased theoretical and application value. However, it still remains unknown whether plant Peps are important for fighting off piercing–sucking insect herbivores, which cause little wounding upon feeding. In this study, we addressed this open question using the rice–BPH interaction as a model and arrived at a positive answer. Our findings revealed OsPep3 as an essential DAMP of rice for defending BPH, and this notion was supported by multiple lines of evidence. Firstly, OsPep3 was transcriptionally induced in rice tissues suffering BPH attacks (Figure 2e). Secondly, OsPep3 elicitation enhanced rice resistance to BPH (Figure 3). Thirdly, disruption of OsPep3 perception by knockout of *OsPEPR*s compromised anti–BPH immunity (Figure 1e–g).

This study further shed insights on the molecular mechanisms underlying OsPep3–induced BPH resistance in rice. The KEGG analysis of OsPep3–upregulated genes in rice leaf sheaths revealed that the phenylpropanoid pathway is promoted by OsPep3. One of the two final products of phenylpropanoid metabolism is flavonoids (Dong and Lin, 2021), which play crucial roles in defending BPH (Dai *et al.,* 2019; Harun–Or–Rashid et al., 2018). Several flavonoids, including naringenin, sakuranetin, schaftoside, isoschaftoside, rhoifolin and apigenin, have been found to be induced by BPH infestation (Uawisetwathana *et al.,* 2019; Xu *et al.,* 2021). However, we failed to detect the induction of these flavonoids by OsPep3. In addition, *OsF3H*, which encodes a key enzyme in flavonoid–oriented phenylpropanoid metabolism (Dai *et al.,* 2019; Dong and Lin, 2021), was downregulated by OsPep3. These results indicated that OsPep3–induced BPH resistance is likely independent of flavonoids.

The other final product of phenylpropanoid metabolism is lignin, a pivotal component determining the mechanical rigidity of plant secondary cell wall (Dong and Lin, 2021). Lignin has also been thought to enhance BPH resistance in rice (Harun–Or–Rashid et al., 2018; He *et al.,* 2020; Zhang *et al.,* 2017). *OsTHT1*, which encodes the hydroxycinnamoyl transferase to channel the phenylpropanoid metabolism into lignin biosynthesis (Dong and Lin, 2021), was upregulated by OsPep3 (Figure 5c). Moreover, the *OsMYB30–OsPAL4* module, which promotes BPH resistance through lignin biosynthesis (He *et al.,* 2020), was also induced by OsPep3 (Figure 5c). These results suggested that OsPep3 elicitation may facilitate lignin accumulation. Notably, two of the top 15 OsPep3–induced genes, namely *OsLTP1*.2 and *OsLTPG2* (Figure S5), were found to encode lipid transfer proteins that participate in the formation of cuticle and suberin (Tapia *et al.,* 2013), two extracellular barriers protecting plants from biotic stresses. Furthermore, the GO analysis of OsPep3–upregulated genes indicated that a large fraction of these genes encode extracellular components (Figure 5d). Taken together, these results indicated that fortified extracellular defence contributes to OsPep3–induced BPH resistance.

The integrated assay of OsPep3–elicited transcriptomic and metabolomic changes in rice leaf sheaths also suggested essential roles of lipid metabolism, especially fatty acids or derivatives, in OsPep3–induced BPH resistance. On the one hand, we found that the JA biosynthetic pathway was transcriptionally upregulated by OsPep3 (Figure 6e) and the abundance of JA was significantly increased after OsPep3 treatment (Figures 4a and 6e). In line with our findings, a recent study has demonstrated that JA biosynthesis can be induced by BPH infestation and disruption of JA biosynthesis or JA–mediated defence pathway impairs BPH resistance (Xu *et al.,* 2021). On the other hand, fatty acids and derivatives were overrepresented in annotated metabolites upregulated by OsPep3. In particular, the unsaturated fatty acid PAME, as the top inducible metabolite by OsPep3 (Figure 6d and Figure S6b), is chemically related to palmitoleic acid that has been documented to have a priming effect on anti–herbivore defence responses in maize when exogenously applied (Li *et al.,* 2016). The fatty acid derivative C1P, as another highly inducible metabolite by OsPep3 (Figure 6d and Figure S6b), has been shown to modulate cell death and immunity in Arabidopsis (Bi *et al.,* 2014). It will be attractive for future studies to investigate whether exogenous application of PAME or C1P also improves BPH resistance in rice.

It should be noted that exogenous application of OsPep3 could activate both antixenosis and antibiosis defence mechanisms, which reduced insect settling and feeding, respectively, to defend BPH (Figure 3c,d and Figure S6), whereas knockout of *OsPEPR1* and *OsPEPR2* failed to exert a prominent effect on insect settling and weight gains (Figures S3 and S4). A plausible explanation for this discrepancy is that the OsPep3–OsPEPR signalling is activated upon BPH infestation but is likely to be subsequently perturbed or weakened by BPH salivary effectors (Cheng *et al.,* 2013a). By contrast, exogenous pretreatment of OsPep3 before BPH infestation not only supplies rice with a high dose of mature elicitors but also maximally induces the defence mechanisms without the perturbation from BPH. These findings underscore the advantages of exogenous application of OsPep3 for rice protection.

Overuse of chemical pesticides is currently a prevailing practice in agriculture to control the damage of insect herbivores and pathogens on crops. However, this practice can quickly induce pesticide resistance in those plant opponents. To circumvent this problem, chemical elicitors have recently been invented, which stimulate crop immunity rather than poisoning the pests or pathogens. For instance, exogenous application of furan carbonylhydrazones or bismerthiazol has been demonstrated to improve anti–BPH immunity in rice (Chen *et al.,* 2018; Zhou *et al.,* 2018). Despite the merits of chemical elicitors, their potential adverse impacts on the biodiversity and human health are not fully predictable. In this study, we demonstrated the potential of OsPep3 as an effective immune stimulator in rice (Figure 2c,d) and other Poaceae crops such as wheat (Figure 7g,h). Of note, OsPep3 has particular advantages over chemical elicitors. Firstly, the eliciting activity of OsPep3 is only limited to the Poaceous family (Lori *et al.,* 2015), which minimizes unwanted ecological disturbance. Secondly, OsPep3 is fully digestible for human beings and raises no environmental and food safety concerns. Most importantly, OsPep3 is effective for stimulating rice immunity against chewing (Shinya *et al.,* 2018) or piercing–sucking insect pests (Figure 3) as well as fungal and bacterial pathogens (Figure 7a–f). A potential limitation of OsPep3 in application may be its short lifespan as a peptide. However, this shortcoming can be overcome by developing OsPep3 derivatives with stabilizing chemical modifications or OsPep3–containing nanoparticles (Yao *et al.,* 2018).

## Methods

### Experimental materials

The rice variety ZH11 (Zhonghua 11) and wheat variety Bainong 207 were used as wild–type plants. Rice plants were routinely grown in a greenhouse with a cycle of 14–h light at 28 °C and 10–h dark at 25 °C. To evaluate the developmental phenotypes, rice seeds of ZH11 or *ospepr1pepr2* plants harvested at the same time were surface–sterilized and placed on 1/2 MS agar medium in a plant growth chamber under the conditions described above. A BPH laboratory strain obtained from the Guangdong Academy of Agricultural Sciences, the *M*. *oryzae* strain Guy11, and the *Xoo* strain PXO99A were used in this study.

### Generation of plasmids and gene knockout plants

The CRISPR–GE web server (http://skl.scau.edu.cn/targetdesign/) (Xie *et al.,* 2017) was used for designing two gene–specific sgRNAs targeting *OsPEPR1* or *OsPEPR2* (*OsPEPR1*–sgRNA1: 5′–TAGCCCTTCTTTCTCTGTCT–3′; *OsPEPR1*–sgRNA2: 5′–GTGCGCTATACCATAGCTCT–3′; *OsPEPR2*–sgRNA1: 5′–GCTCAAACTACAACACAATG–3′; *OsPEPR2*–sgRNA2: 5′–ATCCGCTACAGCATAGCTCT–3′). The binary plasmid *pYL–CRISPR/Cas9–OsPEPR1/2* simultaneously expressing these four sgRNAs was constructed according to the Golden Gate ligation procedure described previously (Ma and Liu, 2016). The binary plasmid was transformed into *Agrobacterium tumefaciens* strain EHA105 cells through electroporation. Agrobacteria containing the plasmid were used for transforming rice callus. Transformed rice callus cells were selected by hygromycin resistance and regenerated into whole plants according to a standard protocol (Hiei *et al.,* 1994). *OsPEPR* knockout plants were identified by Sanger sequencing of the PCR products spanning the target regions in individual transgenic lines.

### Sequence alignment and phylogenetic analyses

Putative plant Pep sequences were obtained by the BLAST analysis (https://blast.ncbi.nlm.nih.gov/) using ZmPep1 (VRRRPTTPGRPREGSGGNGGNHH) as an inquiry sequence. Multiple sequence alignment was performed using the ClustalW algorithm. A phylogenetic tree was generated using the neighbour–joining method via the MEGA software (version 6).

### OsPep treatment

OsPep peptides were synthesized by Xinghao Chemical (Wuhan, China) according to the sequences as follows: OsPep1: ARLRPKPPGNPREGSGGNGGHHH; OsPep2: DDSKPTRPGAPAEGSGGNGGAIH; OsPep3: ADSAPQRPGSPAEGAGGNGGAVH; OsPep7: SKPKPEPPGYPREGGGGNGGVVD. The OsPep solution was freshly prepared by dissolving the peptide powder in distilled water to a working concentration of 10 μm before plant application. The detergent silwet L–77 was added to the OsPep solution to a final concentration of 0.05% to facilitate plant penetration. The initial treatment was conducted by submerging the rice leaf sheath into the OsPep solution for 30 s. After BPH infestation, the leaf sheaths were treated by spraying the OsPep solution every 24 h.

### ROS burst assay

Twelve leaves from 10–day–old rice or 2–week–old wheat seedlings were used to generate leaf discs of 0.25 cm^2^ using a hole punch and were incubated in water overnight in a 96–well plate to eliminate the effects of wounding. The next morning water was replaced by 100 μL reaction solution containing 200 μm luminol L–012 (Wako, Japan) and 1 μg horseradish peroxidase (Sigma, St. Louis, MO, USA) supplemented with 10 μm OsPep plus 0.05% silwet L–77 or silwet L–77 only (mock). The luminescence was recorded by a Varioskan Lux luminometer (Thermo, Waltham, MA, USA) for a period of 30 min.

### MAPK activation assay

Leaves detached from 10–day–old rice or 2–week–old wheat seedlings were treated with 10 μm OsPep plus 0.05% silwet L–77 or silwet L–77 only (mock) for 15 or 30 min. Treated leaves were ground in liquid nitrogen, and the tissue powder was immediately mixed with the 6 × SDS–PAGE loading buffer and heated at 95 °C for 5 min. Total proteins were resolved in a 10% SDS–PAGE, and MAPK activation was evaluated by immunoblotting using anti–pERK antibodies (Cell Signaling Technology, Beverly, MA, USA).

### Protoplast preparation and transfection

Isolation and transfection of rice protoplasts from 10–day–old plants was conducted as previously described (Zhang *et al.,* 2011). Transfected protoplasts were incubated in the dark at room temperature for 12 h for protein expression.

### BPH infestation assay

At the four–leaf stage, rice seedlings were infested with BPH nymphs of the third instar at a density of 15 insects per plant. At indicated day post–infestation, individual plants were given a resistance score of 0, 1, 3, 5, 7 or 9 according to the routinely used scoring criteria (Figure S2) (Huang *et al.,* 2001). For the BPH host choice test, two ZH11 seedlings and two *ospepr1pepr2* seedlings at the four–leaf stage were grown in a single plastic bucket side–by–side. Forty BPH nymphs of the third instar were released at the centre of the plastic bucket. The number of nymphs on each plant was recorded at 48 h post–infestation. The honeydew and body weight of BPH nymphs were weighed according to a previously described procedure (Pathak *et al.,* 1982). Briefly, thirty BPH nymphs of the third instar were weighed and starved for 2 h. These BPH nymphs were then placed in a pre–weighed Parafilm sachet attached to the leaf sheath. The sachets were removed after 48–h infestation. The BPH nymphs and sachets were weighed separately. The weight difference in the sachets before and after infestation was considered as the honeydew weight, while the change in the insects’ weight was considered as the body weight gain.

### RNA–seq and RT–qPCR

Rice leaf sheaths treated with OsPep3 or mock were ground in liquid nitrogen into tissue powder. Total RNA was extracted from tissue powder using the RNApre Pure Plant Plus Kit (TIANGEN, Beijing, China) according to the manufacturer’s instructions. For RNA–seq analysis, total RNA was subjected to transcriptome sequencing at the BGI Group (Shenzhen, China). For RT–qPCR, total RNA was converted into first–strand cDNA using the PrimeScript RT Reagent Kit with gDNA Eraser (Takara Bio, Tokyo, Japan). The qPCR was performed in a LightCycler 96 Instrument (Roche) using TB Green Premix Ex Taq (Takara Bio, Tokyo, Japan). Os18s rRNA was used as the reference gene. The primers used in qPCR are listed in Table S1.

The RNA–seq data were analysed using the FastQC algorithm, trimmed using the Trimmomatic algorithm and mapped to *Oryza_sativa* MSU_7.0 genome via the TopHat2 algorithm. Differential expression analysis was performed using edgeR (Robinson *et al.,* 2010). Differential expression genes (DEGs) were obtained with a cut–off of |log_2_(FC)| > 1, FDR (false discovery rate) < 0.01. FDR is adjusted *P* value using the Benjamini–Hochberg’s approach. Volcano plot analysis of DEGs was done in R using the ggplot2 and ggthemes package. Hierarchical clustering was done in R using the pheatmap v.1.0.12 (Pretty Heatmaps) package. Gene ontology (GO) enrichment analysis of the differentially expressed genes was implemented using the g:GOst function on the g:Profiler webserver (Raudvere *et al.,* 2019).

### Non–targeted metabolite profiling

Approximately 200 mg of frozen rice leaf sheaths were homogenized in liquid nitrogen and subjected to non–targeted metabolomics analysis at the BGI Group. The BGI’s metabolomic software package metaX and metabolome information analysis process were used for the liquid chromatography–mass spectrometry (LC–MS/MS) data processing, statistical analysis, metabolite classification and function annotation. Molecular masses, retention times and associated peak intensities for each sample were extracted from the raw files. To identify differentially produced metabolites, variable importance in projection score of the first two principal variables in a multivariate PLS–DA model was combined with fold change (FC) and Student’s *t*–test of univariate analysis. To facilitate the annotation of detected metabolites, the metabolite information (e.g. accurate m/z and possible chemical formula) was searched against different databases, including HMDB, KEGG, m/z Cloud, PubChem and ChemSpider. Metabolite data sets in positive and negative ionization modes were merged to a single data set following the previously proposed method (Calderón–Santiago *et al.,* 2016). The volcano analysis and hierarchical clustering of differentially expressed metabolites were performed using the MetaboAnalyst5.0 software (https://www.metaboanalyst.ca/MetaboAnalyst/).

### Phytohormone measurements

Phytohormone measurements were conducted as described earlier (Huang *et al.,* 2021). Briefly, approximately 200 mg of frozen rice leaf sheaths was ground in liquid nitrogen. The powdered samples were dissolved in the extraction buffer (isopropanol: deionized water: HCl, 2: 1: 0.002 [v/v/v]) with internal standards (^2^H_5_–JA and ^2^H_4_–SA, Olchemin). After gently mixing samples for 30 min, dichloromethane was added. The samples were shaken at 4 °C for 30 min and subsequently centrifuged at 13 000 *g* for 5 min. The solvent from the lower phase was collected into a fresh tube and dried using a nitrogen evaporator. The samples were dissolved in 60% methanol. Each sample of 10 μL was injected into a C18 column and analysed using the AB SCIEX Triple TOF 5600+ system as described previously (Yuan *et al.,* 2017).

### *M*. *oryzae* infection assay

The fungal isolate was grown on oat meal agar for producing spores. Spores were collected by flooding the fungal cultures with sterile water, and the spore concentration in the suspension was adjusted to 2 × 10^5^ conidia/mL. Punch inoculation was performed as previously described (Li *et al.,* 2017). Briefly, detached leaves from 1–month–old rice plants were pretreated with 10 μm OsPep3 or mock for 24 h. Spore suspension of 5 μL was dropped at two separate spots on each leaf. The leaves were subsequently kept in a culture dish that contained sterile water supplemented with 0.1% 6–benzylaminopurine and OsPep3 or mock. The lesion sizes were photographed and measured using the Image–Pro Plus 7 (https://www.mediacy.com/imageproplus) at 7 days post–inoculation. Fungal biomass was quantified through the DNA–based qPCR assay of the abundance of *M. oryzae* Pot2 DNA against that of rice ubiquitin DNA (Park *et al.,* 2012). The primers used in qPCR are listed in Table S1.

### *Xoo* infection assay

The *Xoo* strain PXO99^A^ was grown on the potato–sucrose medium (HB8712, Hopebiol, Qingdao, China) at 28 °C for 3 days. The bacterial colonies were suspended with sterile water and diluted to OD_600_ of 0.6 for inoculation. Two–month–old rice plants were inoculated using the leaf–clipping method as previously described (Kauffman *et al.,* 1973). OsPep3 at 10 μm or mock was applied by spraying at 12 h before bacterial inoculation and every 12 h after inoculation. Disease was scored by measuring the lesion length 10 days after inoculation at 28 °C. Bacterial growth in rice leaves was measured by counting the colony–forming units (cfu) as previously described (Sun *et al.,* 2004). Briefly, the infected leaves were detached and sterilized with 75% ethanol and then homogenized in sterile water. The bacteria in resulting homogenates were recovered on the potato–sucrose medium and the colonies were counted.

### Statistical analysis

Standard statistical analyses were conducted using the GraphPad Prism 8.0 software, San Diego, CA, USA. For multiple comparisons, the one–way of variance (ANOVA) with the post hoc Tukey test was used. For two–sample unpaired comparisons, Student’s *t*–test was used.

## Conflicts of interest

The authors have filed a patent based on the results reported in this study.

## Author contributions

J.–F.L. conceived and supervised this study. W.S. performed most of the experiments. X.Z. conducted hormone measurements. J.L. assisted the assays of OsPep–induced ROS burst and MAPK activation. K.T. assisted bioinformatics analysis. C.L. assisted the *Xoo* infection assay. W.S., J.–F.L., S.X. and W.Z. analysed the data. J.–F.L. wrote the manuscript.

## Supporting information


**Figure S1** *OsPEPR* knockout does not cause obvious developmental defect in rice.
**Figure S2** The criteria used for calculating the BPH resistance score.
**Figure S3** *OsPEPR* knockout does not increase rice attractiveness to BPH.
**Figure S4** BPH nymphs feed equally on ZH11 or *OsPEPR* knockout plants.
**Figure S5** Peps from plant species of the same family tend to be more closely related.
**Figure S6** OsPep3 treatment reduces the feeding of BPH on rice.
**Figure S7** Top 15 most highly inducible genes by OsPep3 in rice leaf sheaths.
**Figure S8** Top 10 most highly inducible metabolites by OsPep3 in rice leaf sheaths.
**Table S1** The qPCR primers used in this study.

## Data Availability

The raw data of RNA–seq were deposited to the NCBI Sequence Read Archive (SRA) database (access ID: PRJNA766326). The raw data of non–targeted metabolomics were deposited to the EMBL–EBI MetaboLights database (access ID: MTBLS3518).
