# Supplementary material for: Plant elicitor peptide signalling confers rice resistance to piercing‐sucking insect herbivores and pathogens
Source: Plant Biotechnol J. 2022 Feb 18;20(5):991–1005. doi: 10.1111/pbi.13781 (PMC9055822; doi:10.1111/pbi.13781)
Supplement: Supplementary file 1 — Figure S1 OsPEPR knockout does not cause obvious developmental defect in rice. Figure S2 The criteria used for calculating the BPH resistance score. Figure S3 OsPEPR knockout does not increase rice attractiveness to BPH. Figure S4 BPH nymphs feed equally on ZH11 or OsPEPR knockout plants. Figure S5 Peps from plant species of the same family tend to be more closely related. Figure S6 OsPep3 treatment reduces the feeding of BPH on rice. Figure S7 Top 15 most highly inducible genes by OsPep3 in rice leaf sheaths. Figure S8 Top 10 most highly inducible metabolites by OsPep3 in rice leaf sheaths. Table S1 The qPCR primers used in this study. [file PBI-20-991-s001.pdf]

Supporting information

Plant elicitor peptide signaling confers rice resistance to piercing-sucking insect herbivores and pathogens

Wenzhong Shen[1], Xue Zhang[1], Jiuer Liu[1], Kehan Tao[1], Chong Li[1], Shi Xiao[1], Wenqing Zhang[1], Jian-Feng Li[1*]

[1]State Key Laboratory of Biocontrol, Guangdong Provincial Key Laboratory of Plant Resources, School of Life Sciences, Sun Yat-sen University, Guangzhou 510275, Guangdong, China.

*Correspondence: J.-F.L. (lijfeng3@mail.sysu.edu.cn)

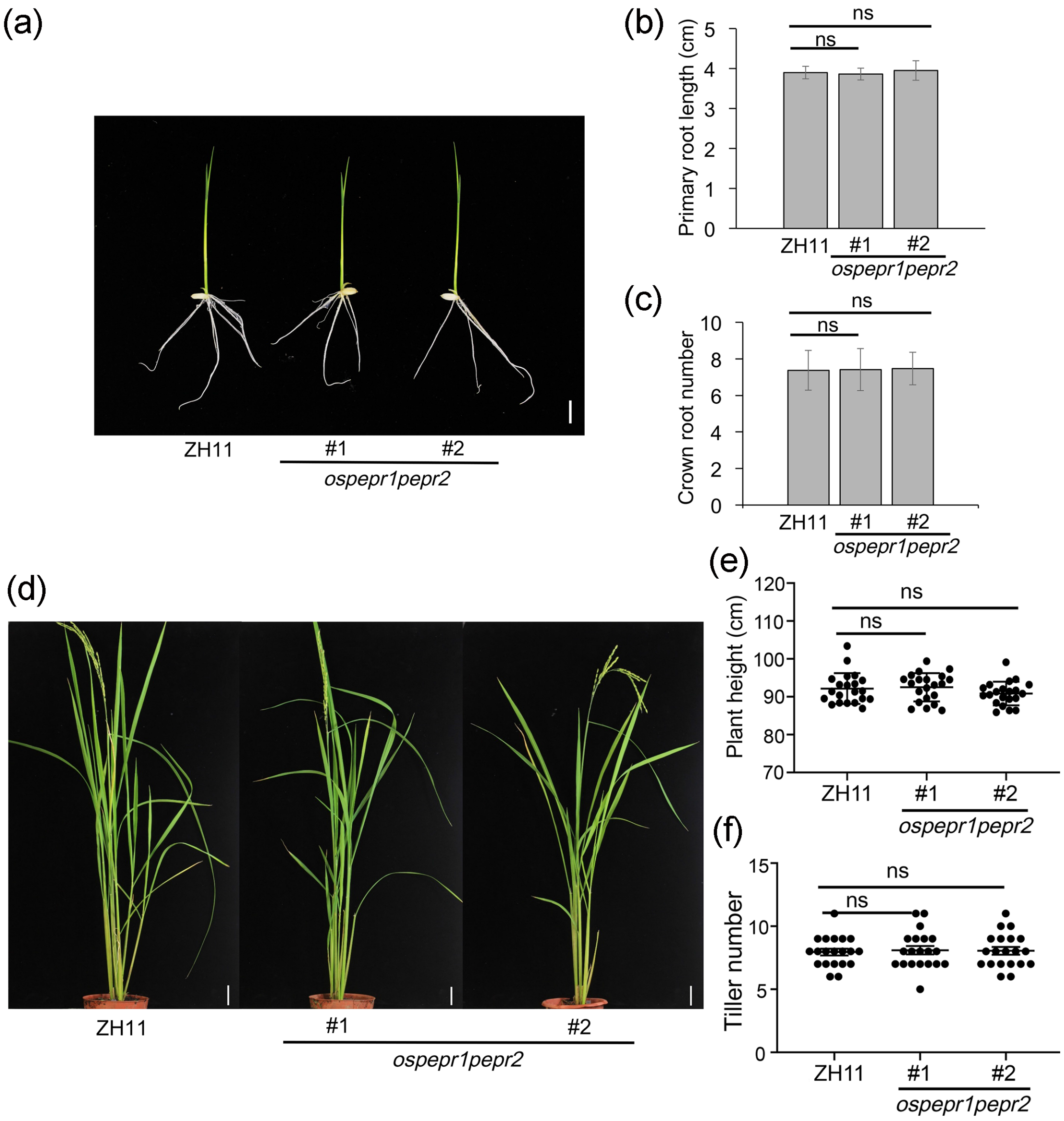

Figure S1. *OsPEPR* knockout does not cause obvious developmental defect in rice. (a) Five-day-old *ospepr1pepr2* null seedlings are indistinguishable from ZH11 seedlings. Scale bar = 0.5 cm. (b) Five-day-old ZH11 and *ospepr1pepr2* null seedlings have comparable primary root lengths. Data are shown as mean ± SD of twenty seedlings with individual data points shown as dots. ns, not significant. (c) Five-day-old ZH11 and *ospepr1pepr2* null seedlings have comparable crown root numbers. Data are shown as mean ± SD of twenty seedlings with individual data points shown as dots. ns, not significant. (d) Two-month-old *ospepr1pepr2* null plants are indistinguishable from ZH11 plants. Scale bar = 5 cm. (e) Two-month-old ZH11 and *ospepr1pepr2* null plants have comparable heights. Data are shown as mean ± SD of twenty plants with individual data points shown as dots. ns, not significant. (f) Two-month-old ZH11 and *ospepr1pepr2* null plants have comparable tiller numbers. Data are shown as mean ± SD of twenty plants with individual data points shown as dots. ns, not significant.

Resistance score	Plant’s physical condition
0	The plant was healthy with intact leaves
1	One leaf of the plant was yellowing
3	One to two leaves of the plant were yellowing or one leaf shrank
5	One to two leaves of the plant shrank or one leaf shriveled
7	Three to four leaves shrank or two to four leaves shriveled, but the plant was still alive
9	The plant was dead

Figure S2. The criteria used for calculating the BPH resistance score.

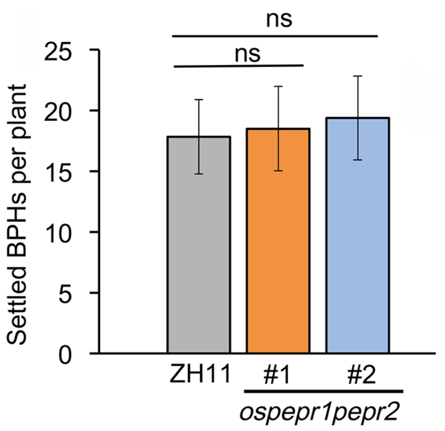

Figure S3. *OsPEPR* knockout does not increase rice attractiveness to BPH. BPH nymphs exhibited no preference between ZH11 and *ospepr1pepr2* null seedlings in a host choice test. The number of nymphs on each plant was recorded at 48 h post infestation. Twenty seedlings were evaluated for each genotype. Data are shown as mean ± SD. ns, not significant.

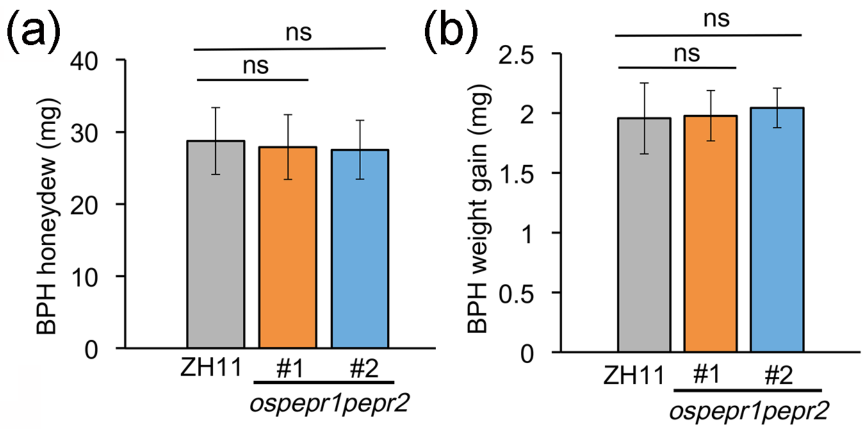

Figure S4. BPH nymphs feed equally on ZH11 or *OsPEPR* knockout plants. (a) BPH nymphs feeding on ZH11 or *ospepr1pepr2* null seedlings produce comparable amounts of honeydew. The honeydew weight of nymphs feeding on each genotype was recorded at 48 h post infestation. Twenty nymphs feeding on each genotype were evaluated. Data are shown as mean ± SD. ns, not significant. (b) BPH nymphs feeding on ZH11 or *ospepr1pepr2* null seedlings have comparable body weight gain. The body weight gain of nymphs feeding on each genotype was recorded at 48 h post infestation. Twenty nymphs feeding on each genotype were evaluated. Data are shown as mean ± SD. ns, not significant.

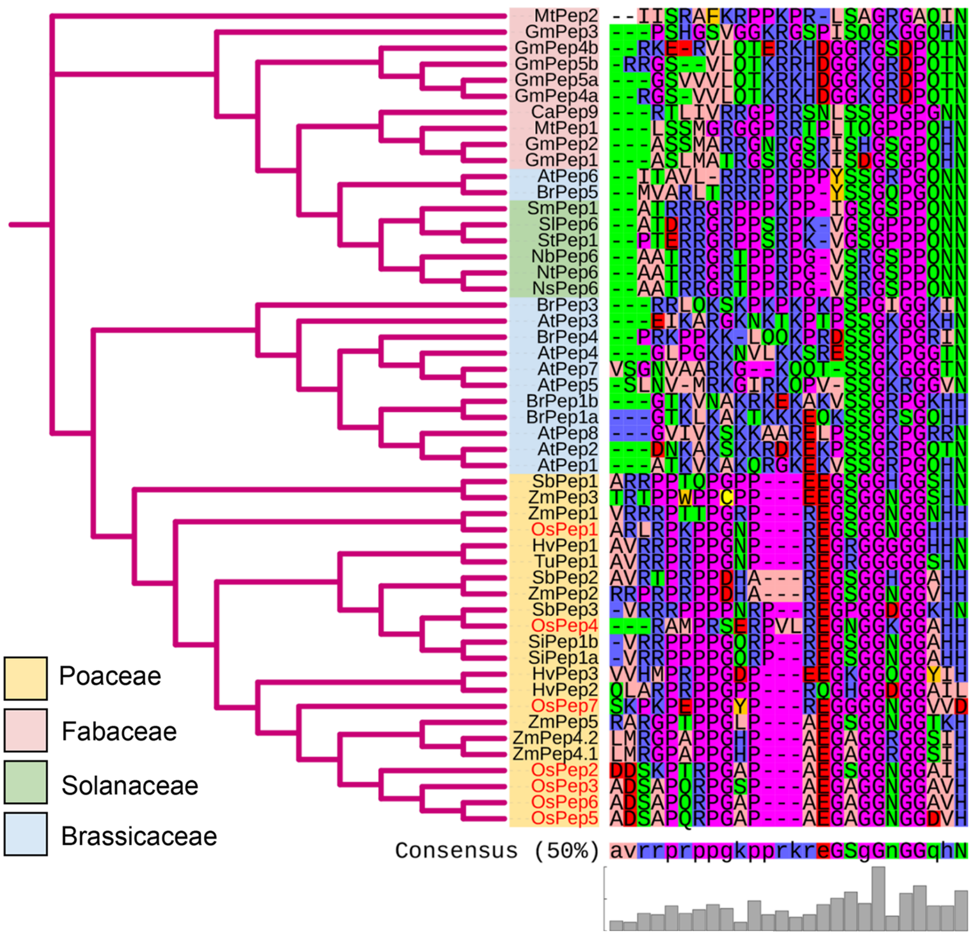

Figure S5. Peps from plant species of the same family tend to be more closely related in sequence. Multiple sequence alignment of putative Peps from 20 plant species in four families was performed using the ClustalW algorithm. Putative Pep sequences were obtained by the BLAST analysis using ZmPep1 as an inquiry sequence. The phylogenetic tree was generated using the neighbor-joining method via the MEGA software.

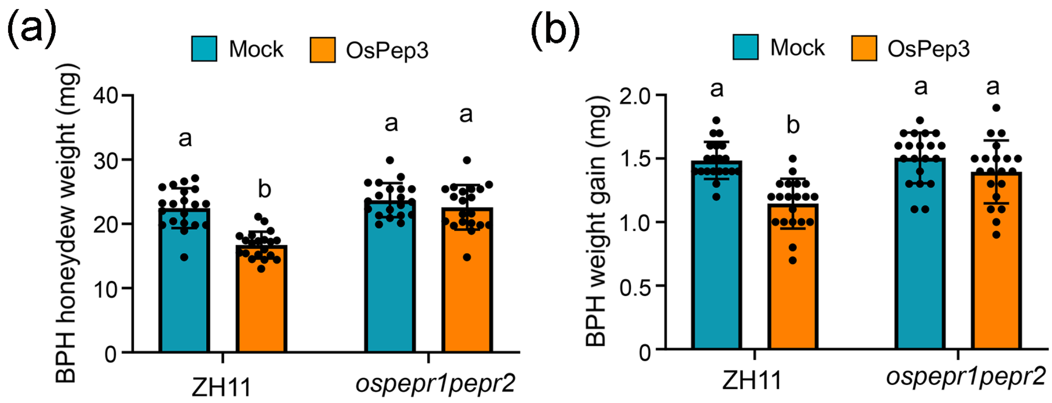

Figure S6. OsPep3 treatment reduces the feeding performance of BPH on rice. (a) BPH nymphs feeding on OsPep3-treated ZH11 seedlings produce less honeydew than those feeding on mock-treated ZH11 seedlings or OsPep3-treated *ospepr1pepr2* seedlings. The honeydew weight of nymphs feeding on each genotype was recorded at 48 h post infestation. Data are shown as mean ± SD of twenty nymphs with individual data points shown as dots. Different letters indicate significant differences with $P < 0.01$ (one-way ANOVA with Tukey's multiple comparisons test). (b) BPH nymphs feeding on OsPep3-treated ZH11 seedlings have less body weight gain than those feeding on mock-treated ZH11 seedlings or OsPep3-treated *ospepr1pepr2* seedlings. The body weight gain of nymphs feeding on each genotype was recorded at 48 h post infestation. Data are shown as mean ± SD of twenty nymphs with individual data points shown as dots. Different letters indicate significant differences with $P < 0.01$ (one-way ANOVA with Tukey's multiple comparisons test).

	Gene_ID	Gene_name	Annotation	Fold change
1	Os04g0107600	*OsADC2*	Arginine decarboxylase	25.33
2	Os07g0526400	*OsPKS15*	Polyketide synthase	17.47
3	Os01g0822900	*OsLTP1.2*	Similar to Lipid transfer protein	15.99
4	Os02g0643200	*OsYABBY4*	Transcription factor	15.52
5	Os03g0830400	*OsFWL-Like*	Unknown function Cys-rich family protein	13.48
6	Os09g0507300	*OsAAO5*	Ascorbate oxidase	13.39
7	Os06g0521400	*OsPRX84*	Haem peroxidase family protein	12.64
8	Os04g0581000	*Os2ODD3*	2-oxoglutarate-dependent dioxygenase 3	12.46
9	Os03g0733600	*OsGIF3*	SSXT family protein	12.07
10	Os06g0604200	*OsPLDα4*	Chloroplast-localized phospholipase D	10.31
11	Os10g0392400	*OsTIFY11d*	Tify domain containing protein	10.27
12	Os03g0830200	*OsFWL6*	Function unknown Cys-rich family protein	10.22
13	Os08g0137900	*OsUCL25*	Similar to Chemocyanin precursor	10.19
14	Os08g0140300	*OsTDC1*	Aromatic L-amino acid decarboxylase	9.35
15	Os03g0167000	*OsLTPG2*	Similar to Non-specific lipid-transfer protein	8.98

Figure S7. Top fifteen most highly inducible genes by OsPep3 in rice leaf sheaths. The induction folds of these genes by OsPep3 were determined by RNA-seq at 24 h post OsPep3 treatment and were shown as mean of two biological replicates.

(a)

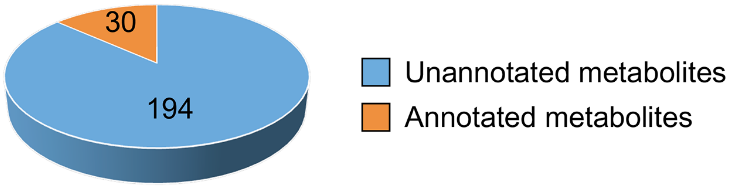

(b)

	Metabolite Name	Fold change	P value	Class
1	Palmitelaidic acid methyl ester	100.88	6.98E-04	Fatty acid
2	Ceramide-1-phosphate	94.76	2.80E-06	Fatty acid derivate
3	Trans-δ2-11-methyl-dodecenoic acid	44.00	9.22E-03	Fatty acid
4	Docosahexaenoyl glycine	39.80	5.73E-06	Fatty acid
5	Linalyl oxide	34.32	5.85E-08	Oxolane
6	Azaspiracid-9	24.69	1.21E-04	Fatty acid derivate
7	Triethyleneglycol bis(2-ethylhexanoate)	24.08	8.57E-07	Fatty Acyl
8	Δ2-trans-hexadecenoic acid	16.76	1.21E-03	Fatty acid
9	Ouabain	7.62	6.42E-04	Steroid derivative
10	Coniine	5.06	4.52E-05	Alkaloid derivative

Figure S8. Top ten most highly inducible metabolites by OsPep3 in rice leaf sheaths. (a) Only 30 out of the 224 metabolites induced by OsPep3 are annotated. (b) Top ten most highly inducible metabolites by OsPep3. Only annotated metabolites were counted. The induction folds of these metabolites by OsPep3 were determined by non-targeted metabolomics profiling at 24 h post OsPep3 treatment and were shown as mean of five biological replicates.

Table S1. The qPCR primers used in this study

q_*OsPEPR1*_F	ACTGGAGTTATTGGCACCTTTG
q_*OsPEPR1*_R	AGTATGACGCCGTAGCTGTA
q_O*sPEPR2*_F	GCATTGCAAAGCTCATGGATCA
q_*OsPEPR2*_R	CACCGTAACTGTACACATCGAAT
q_*OsPROPEP1*_F	TGAAGAGCAGTTATTTCTACATGC
q_*OsPROPEP1*_R	AAGCAGGCTGCCTAGTGATG
q_*OsPROPEP2*_F	CAATGGCCGGAGTACGATCC
q_*OsPROPEP2*_R	AGAAGCAGCGGTGTGGATT
q_*OsPROPEP3*_F	ATGGCATCAGCCTTGGCA
q_*OsPROPEP3*_R	AGGAGGATGGCATTGTGGTG
q_*OsPROPEP4*_F	ATAACACTGCTGCGTCAAGC
q_*OsPROPEP4*_R	TCCCCCGTTCCCTTCTCTTA
q_*OsPROPEP5*_F	TGATCCCCGCACACCTCCTG
q_*OsPROPEP5*_R	ATCACCGGCGGCTGTGGGAG
q_*OsPROPEP6*_F	GACCTCGCCGTCGTCGTT
q_*OsPROPEP6*_R	GGAGCGTCCCTCACCAAC
q_*OsPROPEP7*_F	TGTTGCAGCTGATCCAGCG
q_*OsPROPEP7*_R	GCCTGCAGATTCTCGGTCAC
q_*OsAAO5*_F	CATGCTCGACACCGAGAACA
q_*OsAAO5*_R	GGCCTGTCAACGACGAATCT
q_*OsPAL4*_F	CGTATCCGCTCTACCGGTTC
q_*OsPAL4*_R	GCTTGCCTTCGTTGATAGCC
q_*RBBI3-3*_F	CCAAAGCATCACGACCACGC
q_*RBBI3-3*_R	AACCAGTCACTGCAGACGAAGG
q_*OsMYB30*_F	GTGGCACACTCACCTCAAGA
q_*OsMYB30*_R	GAGGTGAAACTGTCCGTGGT
q_*OsMPKK10.2*_F	ACCCTCCCGCTCGATCACTT
q_*OsMPKK10.2*_R	TTGCCGTGGCCGAGGACGGA
q_*OsTHT1*_F	GATGCGGAACCTCGCCGTCC
q_*OsTHT1*_R	GCGCCGCCGTGATCTTCTTC
q_*Os18srRNA*_F	CTACGTCCCTGCCCTTTGTACA
q_*Os18srRNA*_R	ACACTTCACCGGACCATTCAA
q_*OsUBQ*_F (gDNA)	TTCTGGTCCTTCCACTTTCAG
q_*OsUBQ*_R (gDNA)	ACGATTGATTTAACCAGTCCATGA
q_*MoPot2*_F	ACGACCCGTCTTTACTTATTTGG
q_*MoPot2*_R	AAGTAGCGTTGGTTTTGTTGGAT
